# Simple Quantification of Sticking Propensities of Pharmaceuticals with Mechanochemistry

**DOI:** 10.1021/acs.molpharmaceut.5c00124

**Published:** 2025-05-12

**Authors:** Marta Brocca, Helen Blade, Sten O. Nilsson Lill, Aurora J. Cruz-Cabeza

**Affiliations:** † Department of Chemistry, University of Durham, South Road, Durham DH1 3LE, U.K.; ‡ Oral Product Development, Pharmaceutical Technology & Development, Operations, AstraZeneca Macclesfield, Cheshire SK10 2NA, U.K.; § Data Science and Modelling, Pharmaceutical Sciences, R&D, AstraZeneca Gothenburg, Mölndal 43183, Sweden

## Abstract

Punch sticking poses significant challenges in tablet manufacturing and the need for effective solutions is ever-growing. Direct sticking assessment methods often rely on bulky, material-consuming equipment such as compactor emulators, only available in manufacturing sites, and thus inaccessible for most research labs. Consequently, there only exists limited data on sticking propensities of pharmaceuticals in the literature, significantly limiting our understanding of the issue and how it impacts drug manufacturing. A novel, easy, material-sparing, and lab-friendly method to evaluate sticking trends across diverse systems is presented here. The method employs a mechanochemical technique (ball mill grinding) to measure the materials’ adherence to a stainless-steel substrate (milling ball). After optimization of the operating parameters such as relative humidity pretreatment of materials, a best practice protocol was developed. We measured the sticking propensities of 19 diverse molecular crystalline systems consisting of active pharmaceutical ingredients (APIs), API precursors, and common excipients. The method was effective at differentiating and quantifying the sticking ability of our diverse set of systems, which were classified into low sticking (<30 g/m^2^), medium sticking (30–60 g/m^2^), and high sticking (>60 g/m^2^) propensities. For example, p-nitrobenzoic acid and (R,S)-ibuprofen were found to stick with low propensities to the milling ball (<30 g/m^2^), while D-mannitol was found to stick significantly (>100 g/m^2^). Formulations of the pure materials with microcrystalline cellulose (MCC) were also tested and can be extensively explored with this method. Crucially, the operating parameters of the method (such as the milling times, relative humidity pretreatment of materials, or the material of the milling ball) can be easily adjusted to suit the systems and problem of interest. Our method is robust, nondestructive, and highly versatile and allows for fast quantification of sticking propensities of many systems with small quantities of material. The method has the potential to transform the way we study sticking tendencies of pharmaceuticals, enabling the assessment of sticking propensities significantly early in the development pipeline before manufacturing problems arise.

## Graphical abstract

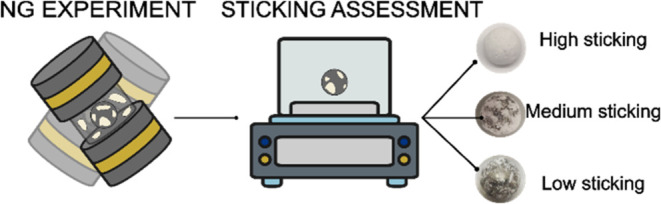

## 1 Introduction

The sticking of materials to the tableting punch is an outstanding issue in the manufacturing of pharmaceuticals. Sticking is a very complex phenomenon, mainly resulting from adhesion interactions between the crystalline particles and the metal surface of the equipment. From a mechanistic point of view, it is generally thought that the forces responsible for sticking comprise van der Waals, electrostatic, and capillary forces.^1–3^ The prevalence of one or more of these forces over the others depends on the crystal structure and polymorphic form of the active pharmaceutical ingredient (API) in the formulation, on its surface energy and surface roughness, as well as on the morphology and size of the crystalline particles.^3–7^ Sticking is also significantly influenced by environmental variables such as temperature and humidity and by the mechanical features and material of the tooling used in tableting. Although the exact mechanisms behind these phenomena remain poorly understood, a simple but effective model (the “Paul–Sun punch sticking model”)^8^ has been proposed to describe it. According to it, punch sticking occurs when the adhesion forces between the punch surface and the API crystals are greater than the cohesion forces between crystals of the API. In the case of formulations, API–punch adhesion forces need to be higher than the API–excipient forces for sticking to occur. This is the first example in the literature in which punch sticking is described in terms of interaction forces. It also describes the three steps involved in tablet sticking,^9^ namely, (a) formation of a monolayer on the metal surface, (b) increase of the monolayer’s thickness, and (c) continuous accumulation of powders on the layer. Depending on the relative strength of the API–API cohesion and API–excipient adhesion forces, the third step may or may not be significant, hence resulting in a more or less severe degree of sticking.

Depending on the severity of punch sticking, final tablets can be defective by either presenting some minor dulling of their surface or more obvious damages like pits around the debossed surface.^10^ Moreover, after a certain amount of compactions affected by adhesion of the material, the tableting tool needs to be disassembled and the punch cleaned and polished.^11^ Defective tablets and unclean punches represent a significant problem in manufacturing because they cause delays in production, reduction in time efficiency, and lowering of the tablet yield, all of these issues ultimately leading to financial losses.

To reduce punch sticking, various strategies have been implemented targeting different aspects of the crystal formulation and the tableting process. Crystal engineering techniques that alter the API crystal structure, along with particle engineering processes, such as increasing the excipient content in the formulation, have proven effective.^12^ Adjusting tableting parameters like compaction pressure can further influence the sticking behavior.^13,14^ Additionally, tooling modifications, including the use of alternative materials for punches and dies, help reduce friction and wear.^15,16^ These combined efforts enhance the efficiency of tablet manufacturing and ensure smoother production processes.

The pharmaceutical industry is not the only one suffering from technological challenges arising from adhesion forces between chemical powders and tooling surfaces.^17^ For example, the accumulation of snacks’ flavoring powders on the manufacturing equipment is a problem for the food industry;^2^ microscopic adhesion of the toner^18,19^ to the surface of ferrite carrier beads impacts the quality of the printed images; or again, the durability and performance of bioceramic-coated bioimplants greatly depend on the adhesion strength between the substrate’s metal and the coating.^20^

Given the impact of this phenomenon on manufacturing efficiency, it would clearly be very advantageous to be able to measure the sticking properties of materials before they reach production using a fast material-sparing method that can be tested early in the R&D process and which can be used to assess batch-to-batch variability. Preliminary experimental assessments of punch sticking, and adhesion in general, are generally carried out with direct measurements using bulky and expensive instruments like tablet compaction simulators,^8,10,13,21–24^ rotary shearing testers^19^ or rheometers.^25^ Many of these techniques require a significant amount of material and are sometimes unsuitable for testing pure systems. Additionally, some methods require custom-built instruments,^2^ making them highly specialized and challenging to replicate.

In this context, the aim of the present work is to design a simple, inexpensive, and small-lab-friendly experimental methodology to quantify the sticking propensity of materials to tool surfaces of different nature. Because the faces of a tablet press are usually made of stainless steel,^1,25,26^ here we also use stainless steel as test material for the development of our methodology. To achieve this goal, the device of choice is a ball mill benchtop instrument, typically used for mechanochemistry and particle size reduction.^27–29^ The intense vibrational motion of the stainless-steel milling jars causes the milling balls to hit and rub against powder samples, breaking the crystals until an equilibrium size is reached (in the nanometer range) typically resulting in the adhesion of particles to the milling ball. Our use of mechanochemistry through the years has shown to us that different materials stick differently to the milling balls. Given this observation, we have developed a simple protocol to quantify sticking of powders to stainless steel in a very simple, efficient, and repeatable way. This simple method can be used to estimate sticking at small scales with very small amounts of material (200 mg), anticipating and preventing problems at later stages of manufacturing. Different tests were carried out to develop a final protocol for the methodology. Several experimental parameters have been optimized, such as the milling time or environmental relative humidities (RHs). The final, optimized protocol has then been applied to a data set of 19 chemically diverse crystalline materials. The advantages and limitations of the method are discussed as well as the sticking data.

## 2 Materials and Methods

### 2.1 Materials

All materials were purchased from either Merck (UK) or Tokyo Chemical Industry (TCI UK Ltd.) or were available in our chemical library and used without any further purification or recrystallization. Powder diffraction was used to identify the starting solid forms. The chemical structures and relevant crystallographic information for all systems are listed in Figure 1. Inorganic salts used for humidity control were purchased from ThermoFisher Scientific (UK). Experiments were mostly performed on the pure systems, but some materials were also mixed with microcrystalline cellulose (MCC, Avicel PH-101) to obtain a 10–90% API–MCC w/w formulation.

**Figure 1:**
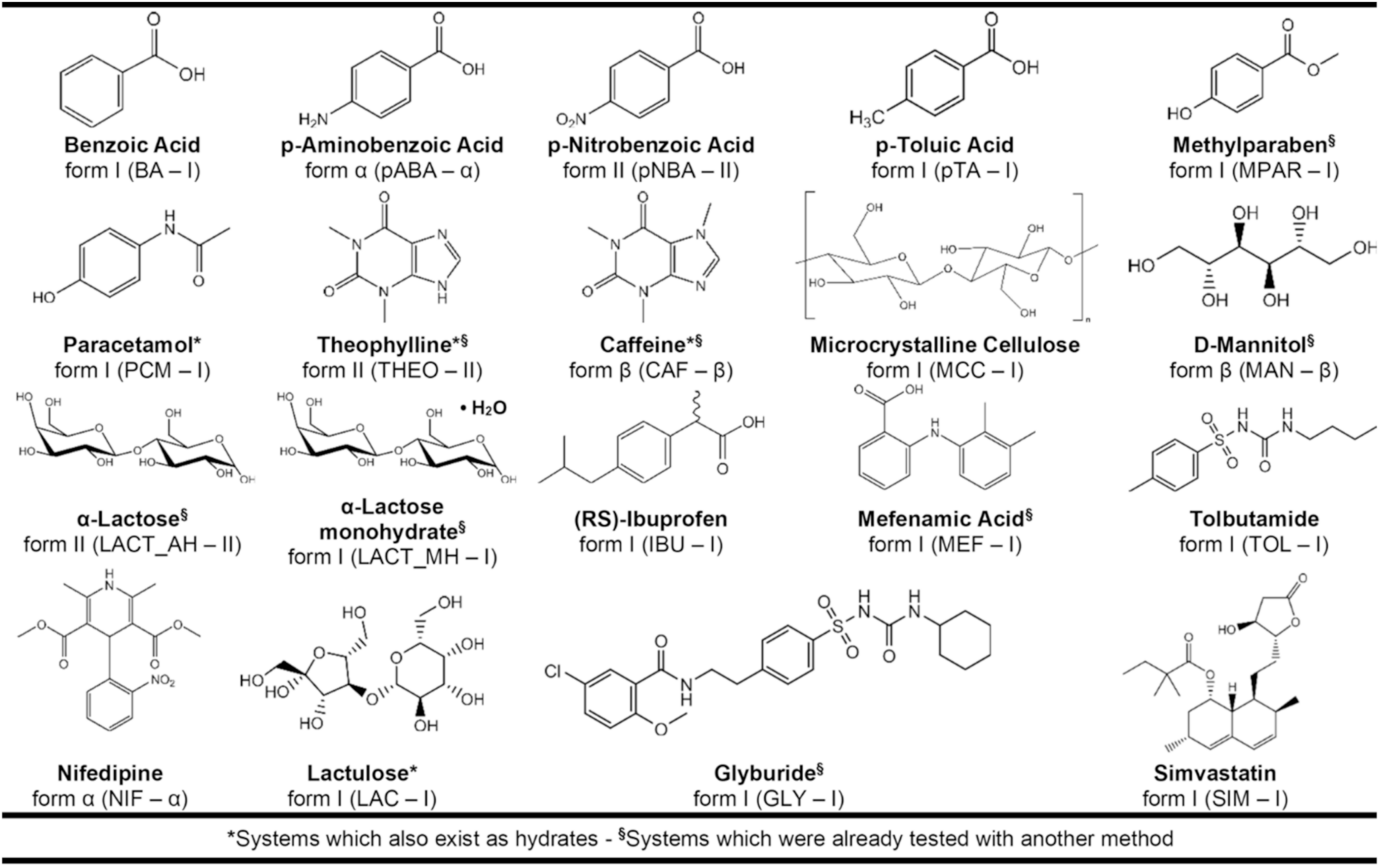
Summary of the 19 systems investigated. Their names are given in bold, followed by their starting solid form and abbreviation.

### 2.2 Experimental Methods

#### 2.2.1 Samples’ Storage under Controlled Humidity at Room Temperature

Prior to any experiments, samples were stored in humidity-controlled environments at ambient temperature (20 ± 5 °C). The crystalline powders of interest were placed into glass vials and stored uncapped in sealed desiccators under controlled relative humidities for at least 48 h. 30, 48, and 73% RH environments were generated using saturated solutions of CaCl_2_·2H_2_O, Mg­(NO_3_)_2_(H_2_O)_6_, and NaCl, respectively.^30^ The RHs inside the desiccators were monitored using two electrical thermo-hygrometers (ThermoPro TP50), reconfirming that the set RH environments were accurately achieved. Whilst the temperature was not controlled, over nearly a year of experiments, our lab temperatures oscillated around 20°C by ±5 °C.

#### 2.2.2 Ball Mill Grinding

Neat grinding (NG) experiments were carried out using an MM400 Retsch Mixer Mill (Retsch Technology GmbH, Haan, Germany) at room temperature. For each NG experiment, ∼200 mg of material was placed in a 5 mL stainless-steel milling jar together with one 7 mm diameter stainless-steel ball. The loaded jars were shaken in the mill for 45 min at a frequency of 30 Hz. For selected materials, several other milling times were also explored (10, 20, and 30 min). For a number of samples, the temperature of the milling jars was recorded immediately before and after the milling had been completed by using a ETEKCITY Lasergrip 1080 infrared thermometer.

#### 2.2.3 Sticking Assessment through a Gravimetric Method

The amount of material adhered to the stainless-steel ball after each NG experiment was measured by using a gravimetric method. The ball was weighed immediately before and after milling, and the weight difference (in g) was used to determine the adhesion of the crystalline powder to the surface of the milling ball. Three independent weighing measurements were done per NG experiment using a Kern ABT 220-5DNM analytical balance (0.01 mg precision).

#### 2.2.4 Sticking Propensity

The sticking propensity of the studied materials was expressed as a *sticking density* (g m^–2^) calculated as the ratio between the mass of the material adhered to the ball (in g) to its surface area (for 7 mm diameter balls, the area is 1.54 × 10^–4^ m^2^). The reported sticking propensities are an average value over three independent NG experiments (per each of the conditions tested) listed together with the calculated standard deviations.

#### 2.2.5 Powder Diffraction

Powder X-ray diffraction (PXRD) patterns of all materials were taken before and after the NG experiments to identify the materials’ initial crystal form and the form obtained upon milling. PXRD patterns were recorded using a Bruker AXS D8 Advance diffractometer, with Cu Kα radiation (λ = 1.5406 Å), in the 2θ 5–55° range, a step size of 0.021°, and a count time of 0.5 s/step. Samples were prepared and mounted on a Si low-background sample holder, and diffraction data were collected immediately after the milling experiment. Crystal forms were identified by comparing the experimental PXRD patterns with those simulated from the relevant structures in the Cambridge Structural Database (CSD).^31^

#### 2.2.6 Searches and Analysis Modules in the Cambridge Structural Database

Neat crystal forms of the systems studied here were retrieved from the CSD (version 5.45) through scripting with the CSD Python API.^32^ A list of the common names of the crystal forms was inputted to a first script which searches the database for organic, single-component, nonpolymeric structures with a maximum R-factor of 5%. The code generates a list of refcodes for each crystal form associated with the input name. After the appropriate forms were selected, their refcodes were entered into another Python script, which identified all structures within the same refcode family and provided the corresponding R-factors for each structure. The crystal structures with the lowest R-factor were chosen. The so-found crystal structures were analyzed with the *Surface Analysis* tool both in Mercury[Bibr ref33] (2024.3.0 version) and with CSD Python API. Surface information on specific *hkl* facets was retrieved, such as the number of hydrogen bond donors and the number of heavy atoms (with an atomic mass greater than 13 amu) on the surface.

#### 2.2.7 Generating Crystal Morphologies

Attachment energy morphologies of the systems were generated with the *Morphology* tool of the Materials Studio software.^34^ Crystal structures were imported into the software and geometry optimizations performed using the *Forcite* module (COMPASSIII^35^ force field with its own charges). Subsequently, a *Growth Morphology* calculation was performed using the same energy model, using a (4, 4, 4) maximum cutoff for the *hkl* indices and a minimum of 0.8 Å for the d^(hkl)^ cutoff. Once the morphology was generated, the visualization tool allowed for the identification of each growth facet. The same facet was visualized in Mercury and analyzed using the *Surface Analysis* tool. All surfaces were generated as a single repeat of the dimensions of the unit cell with the offset adjusted to match the attachment energy output from *Materials Studio*.

## 3 Results

### 3.1 Systems

The sticking propensities of crystalline powders of 19 organic systems were evaluated using the novel methodology presented here. The 19 systems were selected as a representative set of active pharmaceutical ingredients (APIs), API precursors, and excipients covering a variety of chemistries (e.g., acids, bases, hydroxyl groups, etc.) and molecular complexities (e.g., size, flexibility, chirality, etc.). Figure 1 shows the chemical structures of all 19 systems, the crystal forms used in the sticking test, and the corresponding abbreviations. PXRD analysis confirmed the starting (commercial) crystal polymorph for all systems to be the most thermodynamically stable form (according to the literature). Whilst all except one of the materials investigated were anhydrous, five systems (paracetamol, theophylline, caffeine, lactulose, and lactose) are also known to exist as hydrates. The hydrate of lactose was also investigated. Crucially, the sticking propensities of eight of the selected systems had been studied in previous works using a direct compression method^21^ (with the aid of a punch) and thus served as ideal test cases for comparison with our new methodology.

### 3.2 Method Description

Our approach uses ball milling followed by a gravimetric assessment of the milling ball. The approach is similar to large-scale methods such as tableting followed by gravimetric assessment of a removable punch,^36^ but at a significantly smaller scale. Figure 2 summarizes the main steps of our procedure.

**Figure 2:**
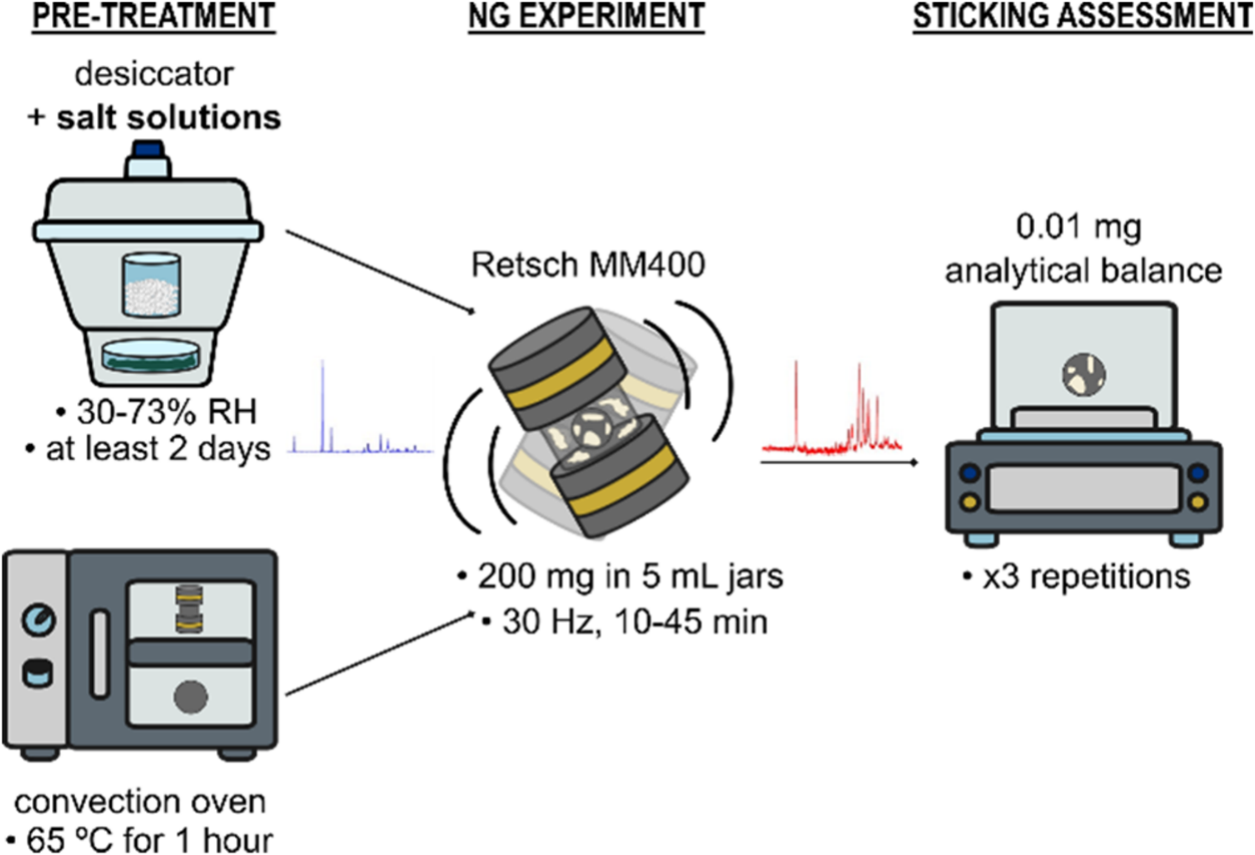
Schematic representation of our new methodology for measuring materials’ sticking propensities.

First, crystalline powders are pretreated by storing them under controlled relative humidity (RH) conditions. A range of RH between 30 and 73% can be explored by changing the saturated salt solutions used to control the humidity environment during the storage. The stainless-steel milling jars and balls are stored in a convection oven at 65 °C for an hour prior to experimentation in order to remove any adsorbed water and then taken out of the oven to room temperature for some minutes prior to the experiment. Second, 200 mg of the pretreated crystalline material are retrieved from the humidity-controlled desiccator and placed into the jar with one ball. The milling jar (with a screwtop) is sealed as quickly as possible and loaded onto the Mixer Mill instrument. The loaded jar is then shaken for the desired amount of time (we explored 10–45 min) at 30 Hz. The crystal form of the material is evaluated before and after milling (typically within 20 min after grinding) with PXRD to monitor any changes in the solid form. Third, after the milling experiment, the milling ball adhered powder on its surface. The ball is taken from the jar and placed on a weighing boat carefully, ensuring that only the powder sticking to the ball is assessed and there is no free-flowing powder. Three independent gravimetric measurements are then taken on an analytical balance. The change in weight of the ball, measured before and after the milling experiment, is used to quantify the sticking mass of the material onto the steel-made milling ball. The sticking density is then calculated by dividing the mass of the material stuck to the ball (in g) by the surface area of the milling ball (in m^2^). To minimize any uptake of moisture during weighing, the three measurements are performed as quickly as possible covering the samples with a glass vial during balance calibration. Finally, the entire procedure is repeated three times, so that an average sticking density across three independent experiments can be derived together with a standard deviation.

### 3.3 Impact of Experimental Variables on the Sticking Propensities

As part of the method’s optimization procedure, we investigated how the sticking densities of selected subsets were impacted by the change in experimental conditions. The variables explored were: (a) relative humidity pretreatment of materials, (b) milling times, and (c) RH value.

#### 3.3.1 Relative Humidity Pretreatment of Materials

First, we investigated the impact of controlling the RH on the sticking assessment of four diverse systems: MAN-β, GLY-I, MPAR-I, and IBU-I. Samples of the four systems were prepared in two ways: (a) with no specific pretreatment and (b) by storing them at 30% RH for 2 days prior to the milling experiments. Sticking propensities with and without humidity pretreatment are presented in Table 1. Measured sticking densities ranged from 19 to 103 g m^–2^ for these four materials. Whilst humidity pretreatment had no impact on the sticking densities of the two less sticky systems (MPAR-I and IBU-I), it significantly impacted the reproducibility of results for the two more sticky systems (MAN-β and GLY-I). We note that our laboratory temperatures generally fluctuate within the range of 20 ± 5 °C, while the RH varies between 44 ± 32%. Pretreating the samples by storing at 30% RH for at least 2 days significantly improves the reproducibility of the sticking densities obtained for MAN-β and GLY-I, lowering the standard deviations of the measurements from 100% in no control to less than 5%. Given this data, it was established that RH pretreatment of materials was a key necessary step of the methodology to enable data reproducibility.

**Table 1:** Sticking Densities of Four Materials with and without Humidity Pretreatment^a^

**material**	**sticking density (g m** ^–**2**^ **)**	
	no pretreatment	30% RH pretreatment
D-mannitol-β (MAN-β)	45 ± 49	103 ± 1
glyburide-I (GLY-I)	50 ± 48	64 ± 3
methylparaben-I (MPAR-I)	33 ± 2	35 ± 4
(R,S)-ibuprofen-I (IBU-I)	17 ± 1	19 ± 3

a Each NG experiment was carried out at 30 Hz for 45 min.

#### 3.3.2 Milling Times

Second, we investigated the impact of milling times on the sticking propensities of six materials at 30% RH. Milling experiments were performed for 10, 20, 30, and 45 min for all six of them (Figure 3). Data in Figure 3 show that milling times do not seem to significantly affect the measured sticking densities for the four least sticky materials: pNBA-II, IBU-I, NIF-α, and PCM-I. For the two stickiest systems (MAN-β and THEO-II), however, the measured sticking densities showed significant fluctuations in their values and appeared to increase with milling times for MAN-β. Looking at the obtained standard deviations, these are higher at lower milling times (10 and 20 min) for all systems. An explanation for this observation may be that homogeneity of the crystalline powder is not achieved at the lower milling times. Upon milling, crystals break (and grow) progressively until an equilibrated distribution of sizes, shapes, and surfaces is achieved.^37^ It is well known that different crystal morphologies of the same crystalline form can have very different sticking tendencies^3,4,6^ since each crystal facet has a different structure and physicochemical properties. As the milling time is increased, the average sticking densities stabilize and they also became more consistent: the 45 min experiment, in particular, gives the smallest deviations in measured sticking values. 45 min is also a standard time^37^ for a milling experiment and led to no solid form conversions in any of the tested systems (see the ESI). We expect that longer milling times may result in some increases in sticking densities in some systems (e.g., MAN-β). Since the standard deviations on the measured sticking densities are low at 45 min of milling, we decided that 45 min was a good choice of the milling time for our methodology.

**Figure 3:**
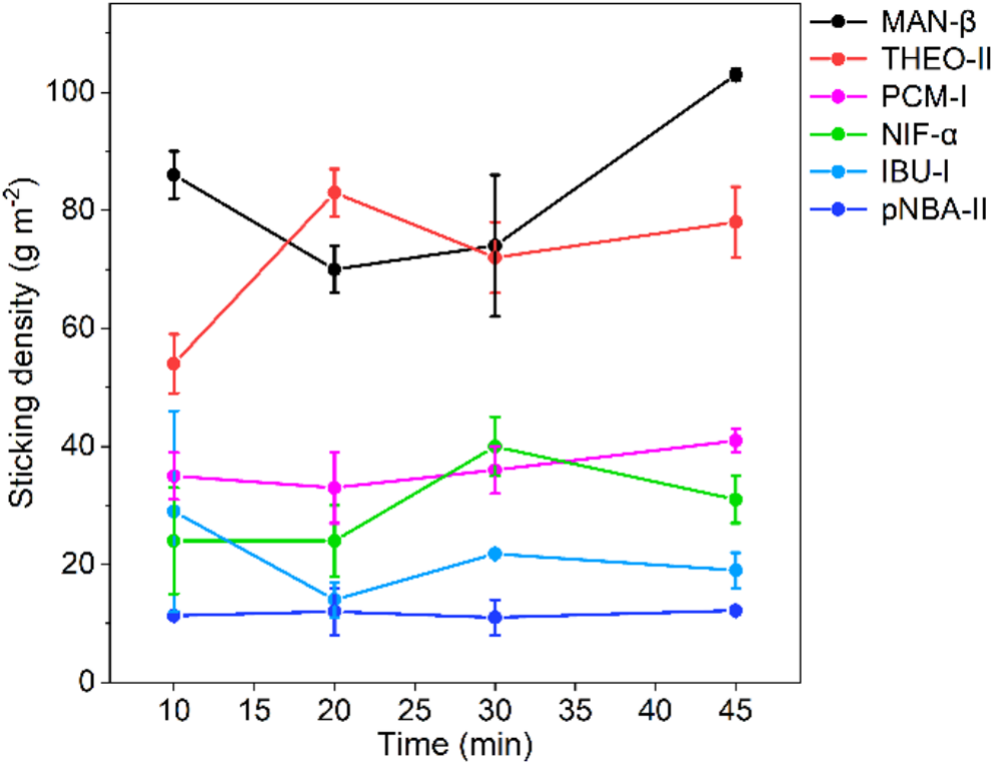
Effect of milling times on the sticking densities of six systems. Each NG experiment was carried out at 30 Hz and 30% RH.

A notable result in these experiments is that the sticking density of MAN-β increases with longer milling times. This result agrees with previous findings^38^ which have shown that, upon milling, the overall surface chemistry of MAN-β is dominated by the new, hydrophilic (011) faces. The increased surface polarity of milled MAN-β is likely to enhance its adhesion to stainless steel, which also contains polar oxide groups.^39^ Similar work^40^ on paracetamol has shown the opposite effect. Upon milling, PCM-I exposes the (010) hydrophobic face (blocking the exposure of the phenolic OH groups on the surface). The predominance of apolar surfaces may lead to lower sticking densities, as observed in our experiments for paracetamol. These observations are supported by other studies on the sticking propensities of mefenamic acid,^3^ celecoxib,^23^ and ibuprofen,^6^ which have shown that high polarity and electronegativity features of crystal surfaces have a significant impact on their adhesion to the metal substrate.

#### 3.3.3 Relative Humidity Value

Above, we have shown that humidity pretreatment is important in the methodology to ensure reproducibility of the sticking densities. Here, we investigate the impact of the RH value on the sticking densities for our six model systems. Milling experiments were performed for 45 min and at 30 Hz with materials pretreated at three different relative humidities (30, 48, and 73%) and PXRD analysis confirmed no changes in solid forms (see the ESI). Sticking densities as a function of RH values for all six systems are listed in Figure 4. Data shows that whilst the measured sticking densities remain similar at all RHs investigated, the standard deviations increase significantly at high RH values, especially for the two highest sticking systems, THEO-II and MAN-β. The large deviations in the results between the three experimental repetitions can be hypothesized to be due to various factors. For THEO-II, we note that 73% RH is above the critical RH required for hydrate formation (60%).^41^ Whilst we observe no evidence of hydrate formation with our PXRD characterization, it is possible that undetectable amounts of theophylline’s monohydrate may exist, hence impacting the sticking properties. For MAN-β, which has multiple −OH groups, it is possible that milling at higher RHs exposes more polar surfaces, leading to more variability in the results.

**Figure 4:**
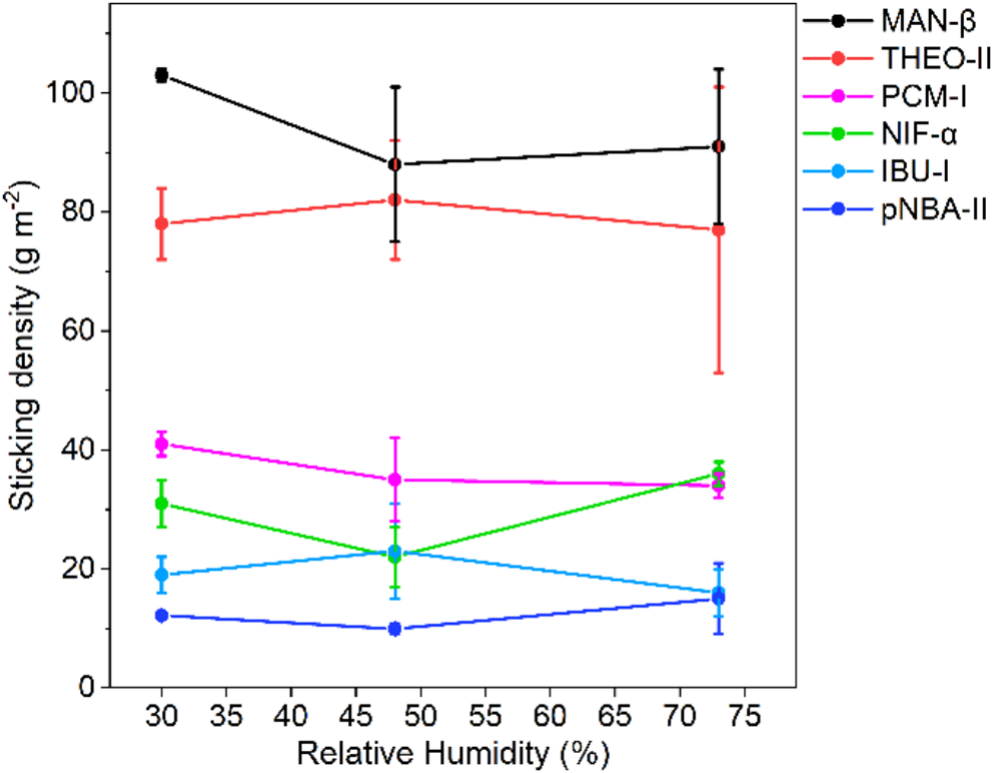
Effect of RH on the sticking density of the six materials. The sticking densities have been obtained after a 45 min/30 Hz NG experiment.

Overall, the data shows that RH can impact the sticking propensity of materials in various ways in agreement with prior work.^42^ Previously, it has been shown that water evaporating from the API powders and tooling can act as a “glue” between the tablets and the punch due to an increase in capillary interaction forces.^43,44^ At the same time, moisture at high RHs can be adsorbed onto surfaces of some crystalline samples, causing an increase in the charge dissipation rate (i.e., the materials are less able to hold the surface charge), hence reducing the sticking.^7,45^ Whilst our method can measure sticking densities at any desired RH, we chose to work with 30% RH since it provides better repeatability.

### 3.4 Comparison of Our Sticking Data to Removable Upper Punch Data

After investigating and establishing the most optimal environmental conditions for the reproducibility of our methodology (Section 3.3), we sought to compare our new sticking data with existing data measured using the well-established “removable upper punch” sticking method.^29^ Since we do not have access to a compaction emulator (typically available in manufacturing sites), we relied on previously reported data on punch sticking to validate our new lab-scale methodology.

To this effect, we used the data published by Paul et al.^21^ who reported punch sticking data for 24 compounds measured using a “removable upper punch tip” method. Because of the variability of operating conditions for these sticking experiments found across the literature and how this impacts the overall data, this study was the best choice for our method validation since, to the best of our knowledge, it reports the largest dataset of sticking data using the removable upper punch method under identical operating conditions. Whilst 24 compounds of pharmaceutical interest were studied, only the structures of 16 of them were reported with the remaining eight being proprietary systems. The study used direct compression of formulations consisting of 10 wt % compound of interest together with 89.75 wt % MCC and 0.25 wt % magnesium stearate using a compactor emulator (PressterTM, Metropolitan Computing Corp., East Hanover, NJ) adapted with a removable upper punch fitted with a 12.7 mm diameter flat-faced tooling.^21^ The upper punch was removed after 100 compactions (25 ms dwell time), and the weight of the adhered material was recorded. Compaction pressures of 100 and 200 MPa were used, and materials were pretreated at 33% RH for 48 h prior to compaction.

For our validation exercise, we purchased those compounds studied by Paul et al.^21^ which were accessible to us commercially and affordably (eight such systems), prepared comparable formulations (10 wt % compound with 90 wt % MCC), pretreated them at 30% RH for a minimum of 48 h, and subjected them to our milling methodology. This ensured that both method conditions and materials are as comparable as possible for the validation. Our sticking densities were compared to those reported by Paul et al. at 100 MPa by converting the mass sticking data into a density (normalized by the surface area of the sticking tool). Similar correlations can be derived at a pressure of 200 MPa.

Sticking densities obtained by Paul et al.^21^ with the removable punch method and our ball mill method are compared in Figure 5 for eight systems. We observe an excellent linear correlation (*R* ^2^ = 0.903) between both methods with significant differences in the absolute values of the sticking densities. Absolute values of the sticking densities are, of course, very much dependent on the specifics of the methodologies. With our ball mill method, we obtain densities two orders of magnitude higher than with the punch tip method. This is due to the significant differences in the geometries and features of the two stainless-steel substrates and the differences of the specifics of the experiment (punching of 100 tablets versus a significant number of impacts of the same ball with the same powder). The sticking trends, however, are extremely well correlated, demonstrating the validity of our method to probe and anticipate punch sticking at a much smaller scale with the more accessible ball milling instruments. We note that whilst the variability of some of our data is significant for some systems (especially MAN-β), no data on variability is reported in Paul’s study,^21^ making it impossible to compare the deviations of the methodologies. The average measured values, however, correlate extremely well (Figure 5) and hence highlight that our simple lab-scale sticking assessment method presented here can reliably anticipate punch sticking issues of APIs and their formulations.

**Figure 5:**
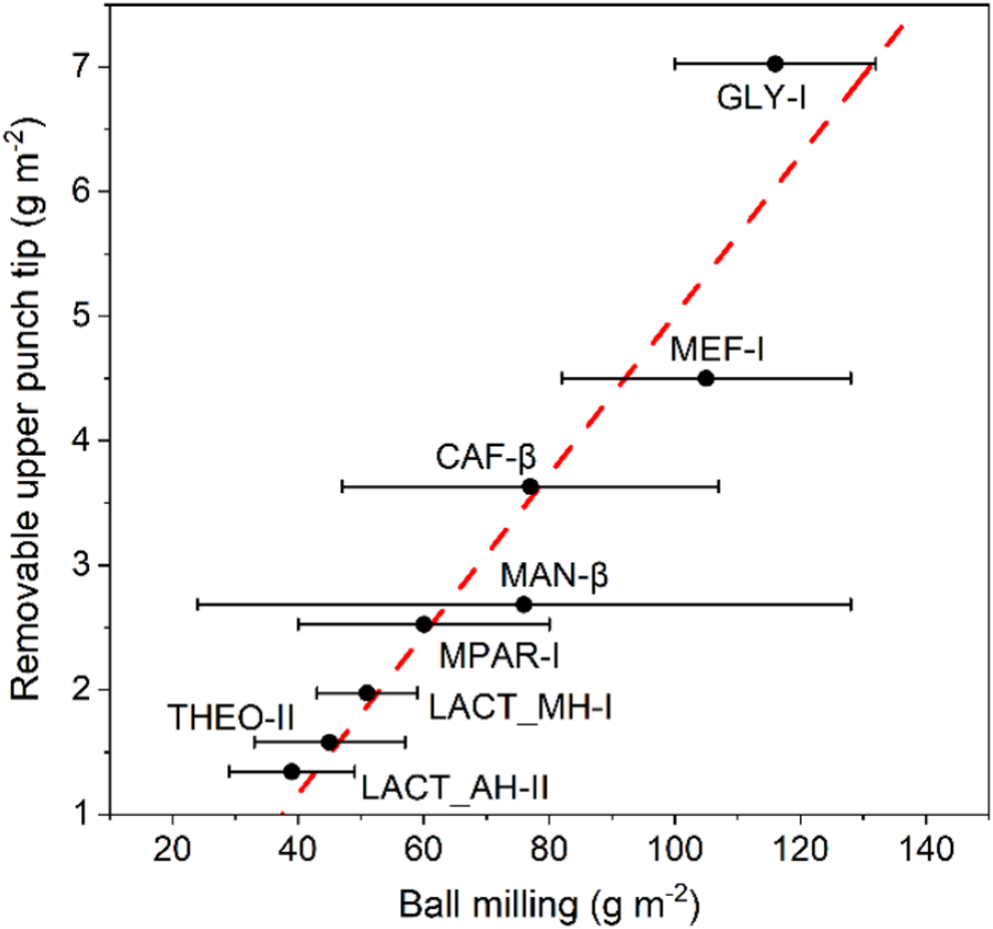
Comparison of sticking propensities (in g m^–2^) of 10–90% (w/w) API–MCC formulations obtained with our ball milling method (present work) and the “removable upper punch tip” method by Paul et al.^21^ The NG experiments were carried out for 45 min at a 30 Hz frequency with materials pretreated at 30% RH.

### 3.5 Sticking Densities of Pure Materials versus Formulations

Quantifying the sticking density of the pure crystalline API is desirable since it can help us develop links between the API structure and properties such as the sticking propensity. The measurement of such data for a significant number of API systems can then enable the development of predictive methods to anticipate sticking issues during manufacturing. Medicines are usually developed as formulations rather than as pure chemical substances.^46^ Here, we test the sticking propensities of pure systems as well as formulations for 11 systems and compare them. As a model formulation, we have chosen a mixture of 10% API with 90% microcrystalline cellulose (MCC) w/w to mirror Paul’s study.^21^ All experiments were performed by storing 200 mg of the pure materials or formulations at 30% RH before the NG experiment, which was carried out at 30 Hz for 45 min. A comparison of the measured sticking densities and their standard deviations (error bars) of pure materials and their formulations is given in Figure 6. The data are shown together with the identity function (a red dashed line) as a guide. We expected the sticking density values to be similar between the pure APIs and the formulations since previous works have established (by dissolving and analyzing with UV–vis the material stuck into the removable punch used to compress formulations) that most punch sticking issues typically arise from the API itself rather than the excipient in the formulation.^7^

**Figure 6:**
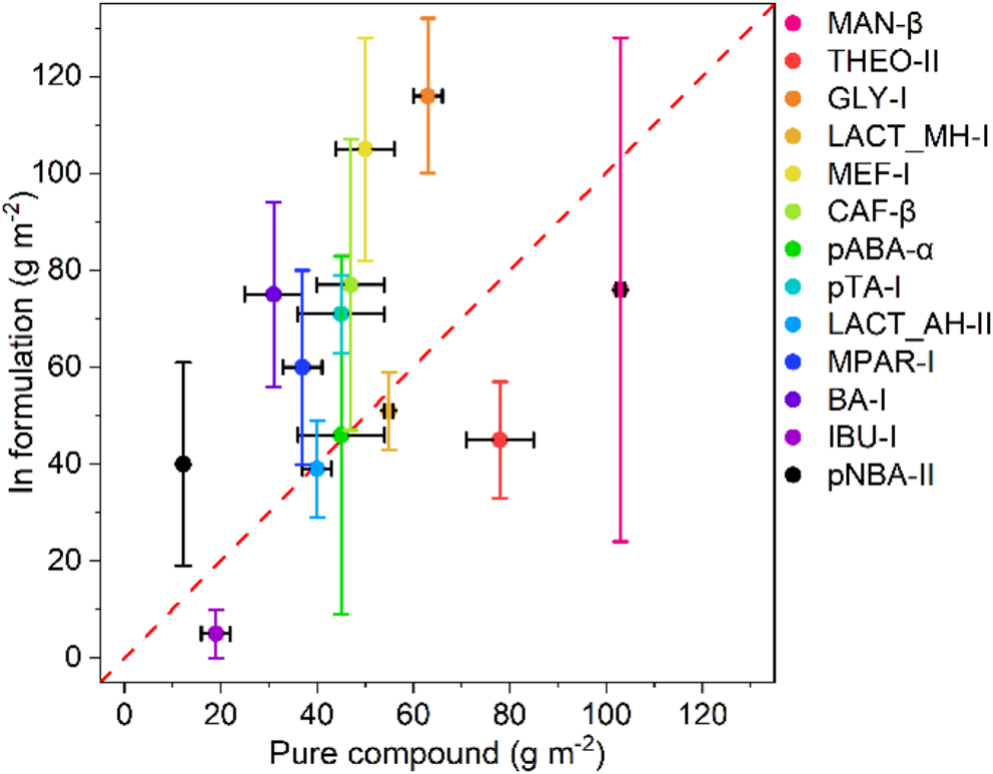
Comparison of the sticking densities of some materials tested pure (*x*-axis) and in formulation with 90% (w/w) microcrystalline cellulose (MCC, on the *y*-axis). The horizontal error bars represent the standard deviation of the NG experiments carried out on the pure materials, and vertical bars correspond to the errors of the experiments with the formulations. All NG experiments were carried out for 45 min, 30 Hz frequency, and 30% RH.

Compared with pure systems, formulations led to higher sticking densities with larger deviations. In that regard, we note that MCC is a very fine powder, and thus the weighing of the formulations becomes more challenging. Loose powders can easily slip from the milling jars onto the weighing boat during the weighing step of the method, interfering with the measurement and leading to higher values and larger errors. This issue is intrinsic to the methodology and can only be minimized by exercising great care when removing the ball from the jar. Moreover, MCC is known to be a hygroscopic material whose mechanical properties are affected by moisture when the RH is around 30% or higher.^47^ For example, the uptake of moisture from MCC increases its plasticity, which in turn is known to increase the stickiness of the material.^21,23^ It is worth highlighting that whilst sample pretreatment is carried out at 30% RH, handling of materials during parts of the procedure was done at environmental conditions, which cannot be controlled. These observations could explain both the higher sticking values for formulations (compared to the pure APIs) and the poorer repeatability of the measurements. For this reason, it would be advisible to either perform the sticking assessments on formulations pretreated at lower RHs (< 30%) or on pure materials only. Alternatively, a stricter control of the environment’s relative humidity can be incorporated into the methodology by, for example, conducting all steps of the assessment in an anaerobic chamber.

Given the limitations of the methodology and the more reliable data obtained for pure systems, we focused our experiments on pure APIs. Previous studies have shown that during punch sticking assessments, 90% of the material adhering to the punch tip corresponds to the API rather than the excipient.^8^ Additionally, it has been demonstrated that the electrostatic properties, which significantly influence the sticking behavior, are primarily driven by the characteristics of the APIs.^48^ Measuring the sticking propensity of the API alone is, therefore, a realistic approach that avoids the challenges related to weighing free-flowing particles under uncontrolled humidity conditions.

### 3.6 Sticking Propensities of 19 Molecular Systems

After optimization of operating parameters and method validation, the final protocol was applied to a large set of 19 organic crystalline materials (see Section 3.1). All milling experiments were performed on the pure systems (after pretreatment at 30% RH for at least 48 h) for 45 min at 30 Hz. Sticking densities for all systems are reported in Table 2 and are plotted in Figure 7. Materials were grouped into three categories: low, medium, and high sticking propensities (abbreviated as LSP, MSP, and HSP) for sticking densities below 30 g m^–2^, between 30 and 60 g m^–2^, and above 60 g m^–2^, respectively. The classification of the sticking behavior into LSP, MSP, and HSP is helpful from a practical perspective and aids interpretation. In LSP systems, adhesion forces are weak and thus crystallites of the materials do not stick to the milling ball. In HSP systems, by contrast, both cohesive and adhesive forces are strong, resulting in the material sticking to the ball and to itself, thus leading to high sticking densities. In MSP systems, adhesive forces are stronger than cohesive forces resulting in variable accumulation of the material on the milling ball. Interestingly, and in line with this interpretation, MSP systems have the largest deviations in the measurements between independent experiments (Table 2). Differences in the behavior for these three categories could be appreciated visually judging from the degree of the materials’ coverage of the milling ball, as shown in Figure 7 for *p*NBA-II, *p*ABA-α, and MAN-β. For these same materials, the variations of surface coverage in the same experiment are shown in Figure S3. MAN-β has the highest sticking density, while *p*NBA-II is the least sticky material of all of the data sets. The fact that MAN-β turned out to be the highest sticking material is noteworthy; it is in fact a very widely used excipient in pharmaceutical industries due to its many advantages over other excipients like MCC and lactose.^49^

**Figure 7:**
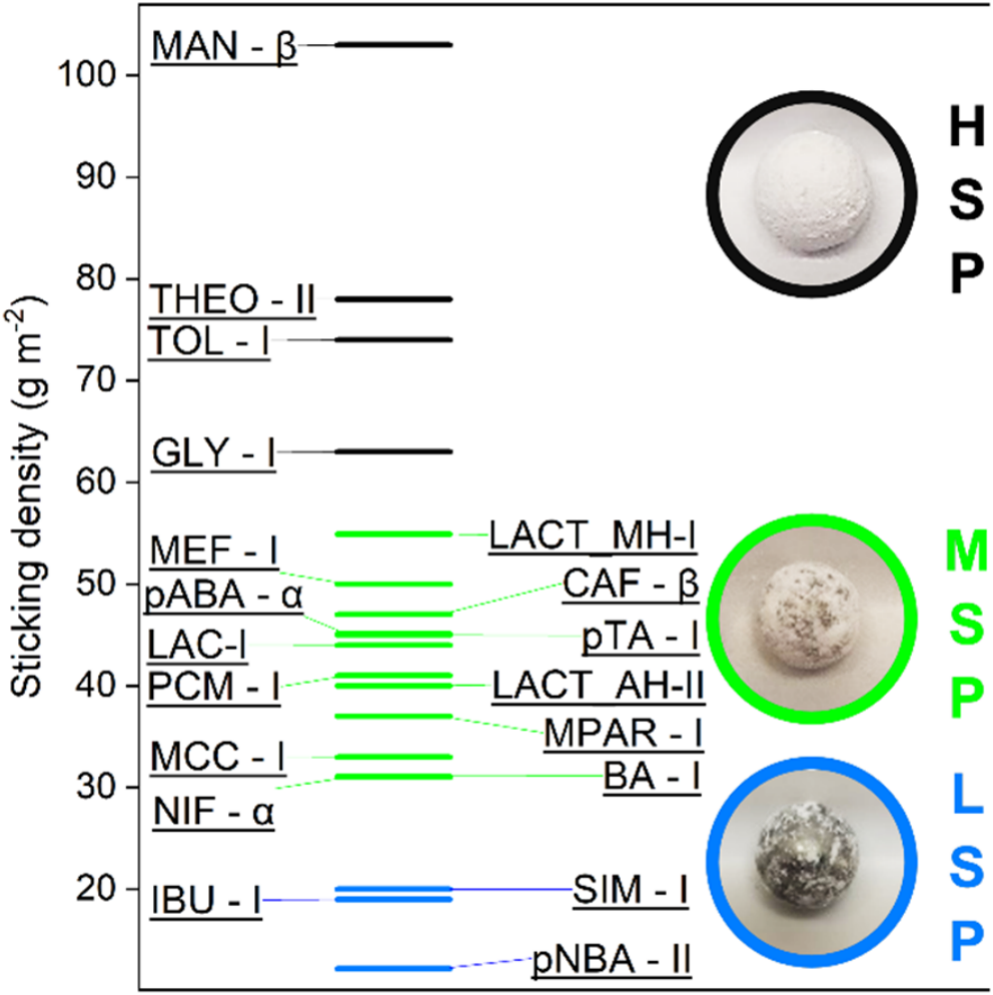
Sticking densities for the 19 studied crystalline molecular systems. NG for 45 min at 30 Hz and 30% RH. Images of the material stuck on the milling ball are shown for D-mannitol-β (HSP), *p*-aminobenzoic acid-α (MSP), and *p*-nitrobenzoic acid-II (LSP).

**Table 2:** Sticking for 19 Diverse Crystalline Systems Evaluated with Our New Ball Milling Method (NG for 45 min at 30 Hz and 30% RH)^a^

**class**	**system**	**REFCODE**	**space group**	**sticking mass (mg)**	**sticking density (g m** ^**‑2**^ **)**	**melting point (°C)**	***T***_**g**_ **(°C)**	**refs**
**HSP**	MAN-β	DMANTL15	P2_1_2_1_2_1_	15.9 ± 0.2	103 ± 1	167	13	^50,51^
	THEO-II	BAPLOT07	Pna2_1_	12.0 ± 1.0	78 ± 7	272	94	^51,52^
	TOL-I	ZZZPUS02	Pna2_1_	11.3 ± 0.6	74 ± 4	128	12	^53,54^
	GLY-I	DUNXAL01	P2_1_/n	9.8 ± 0.5	63 ± 3	172	62	^51^
**MSP**	LACT_MH^b^-I	LACTOS12	P21	8.5 ± 0.1	55 ± 1	214	-	^55^
	MEF-I	XYANAC08	P1̅	8.0 ± 2.0	50 ± 11	230	51	^51^
	CAF-β	NIWFEE03	Cc	7.0 ± 1.0	47 ± 7	236	72	^51,56^
	pABA-α	AMBNAC19	P2_1_/n	7.0 ± 1.0	45 ± 9	188	-	^57^
	pTA-I	PTOLIC01	P1̅	7.0 ± 1.0	45 ± 9	180	-	^58^
	LAC-I	BOBKUY10	P2_1_	6.8 ± 0.8	44 ± 6	162	88	^59^
	PCM-I	HXACAN28	P2_1_/n	6.3 ± 0.4	41 ± 2	170	25	^60,61^
	LACT_AH^b^-II	EYOCUQ01	P1	6.2 ± 0.5	40 ± 3	195	105	^51^
	MPAR-I	CEBGOF01	Cc	5.7 ± 0.7	37 ± 5	126	-	^62^
	MCC-I	-	-	5.1 ± 0.8	33 ± 5	157	-	^63^
	BA-I	BENZAC12	P2_1_/n	4.7 ± 0.9	31 ± 6	123	-	^64^
	NIF^b^-α	BICCIZ05	P2_1_/c	4.7 ± 0.6	31 ± 4	168	45	^51,65^
**LSP**	SIM-I	EJEQAL	P2_1_2_1_2_1_	3.0 ± 0.5	20 ± 3	137	35	^61,66^
	IBU-I	IBPRAC21	P2_1_/c	2.9 ± 0.5	19 ± 3	80	–45	^67,68^
	pNBA-II	NBZOAC16	P2_1_/n	1.9 ± 0.1	12 ± 1	240	-	^69^

a Systems are ranked from the highest to lowest sticking densities.

b Amorphization is observed.

## 4 Discussion

### 4.1 Discussion on the Methodology

Regarding our sticking methodology itself, it is worth discussing several caveats since sticking is a complex process that may be impacted by many variables such as molecular structure, crystal structure, and environmental operational parameters.

Regarding crystal size, the effect of particle size on the sticking of pharmaceutical materials to the tablet punch tooling has been investigated in the past; however, existing data are very limited, and results have been inconclusive. Paul et al. reported that some pharmaceutical systems stick more to the punch as the particle size of the API in the batches increases whilst others stick less.^70^ This was studied for formulations containing crystals of APIs with typical sizes between 5 and nearly 200 μm. Statistical models applied to the understanding of sticking have also led to data pointing toward particle sizes having a minor effect on sticking in pharmaceuticals.^21^ To complicate things further, the crystal shape may be even more important than size since by varying crystal morphologies, different facets with different chemistries are exposed for the same API system.^5,6^ In our method, crystals are broken in the mill until they reach a steady-state crystal size and shape,^37^ typically in the order of 50–100 nm^71–73^ under neat grinding conditions as it has been previously shown. At those small particle sizes, there is the expectation that the crystals will stick more, and our sticking densities are ∼10 times greater than those measured through punch sticking after 100 compactions. It is difficult to ascribe, however, greater sticking to particle size rather than the experiment itself, which uses the mechanical agitation of the ball within the milling jar, which rubs against the molecular crystals and the walls of the balls at 30 Hz. Our sticking data of nm crystals using mechanochemistry correlate very well with the data reported on sticking of μm crystals using the removable punch method (Figure 5), reinforcing that the trends across diverse systems studied under the same conditions remain, even at different length scales and using different sticking methods. Because the steady-state particle size and shape of each system upon milling shall always be the same^37,72,73^ (since milling leads to equilibrium particle sizes), this eliminates the variability of results due to particle size and shape effects for each system studied with our method. Hence, independent studies using the same methodology should all measure the same sticking density since the milling procedure under the same milling conditions leads to the same steady-state particle size and shape. However, this steady-state size and shape will vary across the different systems studied, typically between 50 and 100 nm.

The milling experiments are all carried out at the temperature of our lab, which typically oscillates around 20 °C by ±5 °C. The temperatures of the milling jars were measured before and after milling of several samples with a typical 1 °C increase observed. Our ball milling procedure does not significantly increase the overall temperature of the samples. This agrees with previous works by Kulla et al.^74^ who monitored the temperature variations of samples within a milling jar in situ with the aid of a Raman probe, concluding that the mechanical impact of the ball results in only a minimal increase in temperature. Local temperatures where the mechanical impacts occur, however, may indeed be higher and are difficult to quantify experimentally. In our case, the average overall 1 °C increase in temperature of the milling jar (recorded ex situ) is smaller than the temperature variability of our lab and remains significantly below the melting temperature of the materials studied (with most materials melting between 120 and 280°C with the exception of IBU-I, Table 2). Consequently, our operating milling conditions do not result in local melting of the materials though they will impact the particle size, and it may lead to amorphization in some cases.

The solid-state structure must also influence the propensity of the API to stick to surfaces. In our methods, crystal form changes were monitored by doing PXRD characterization of materials before and immediately after milling (measurements taken within 20 min maximum). We did not observe any conversion of crystal forms and we only observed amorphization for NIF-α, LACT_AH-II, and LACT_MH-I lactose systems (Figure S4). The amorphization is confirmed in these samples by the disappearance or significant broadening of most diffraction peaks as well as the appearance of a broad bump in the diffraction pattern (Figure S4). Amorphization of pharmaceuticals under ball milling at room temperature is dependent on a number of factors including the intensity of the milling, the time of milling, and crucially the glass transition temperature (*T*_g_) of the material under study as well as the RH conditions of the experiment (since water acts as a plasticizer^75^). Milling above the *T*_g_ will never result in amorphization. For about 60% of our systems, their *T*_g_s lie above room temperature, and thus amorphization through milling may be possible. However, milling intensities in ball mills are significantly lower than in planetary mills, our milling times are low (45 min), and there is some humidity in our milling conditions (materials pretreated at 30% RH). Consequently, no amorphization was observed for most systems under investigation. Changes in the intensity and shape of some diffraction peaks for MAN-β, pNBA-II, and THEO-II were observed after milling. These are due to the reduction in particle size that occurs during milling, which eliminates the preferred orientation phenomenon commonly observed for the larger particles of the starting materials.

Finally, balls made of different materials can be used with this method to evaluate the sticking propensity of APIs to other materials. Specifically, the instrument used for these experiments could have also been equipped with stainless/hardened steel, tungsten carbide, agate, zirconium oxide, or PTFE jars and a milling ball, which can all be purchased from the manufacturer. The decision of choosing stainless steel for the current work was made because of the instrument’s availability in the lab and also because stainless steel is the material typically used in tablet punches. Additionally, it is important to note that the size, shape, and number of the milling balls can affect the energy transfer and the degree of particle size reduction, thus influencing the sticking behavior. The ability to easily modify the experimental setup is a major advantage of this method because it can potentially be adapted to investigate multiple adhesion scenarios.

### 4.2 Discussion on Sticking Propensities of the Studied Systems

Different properties of the crystalline materials, including molecular structure, packing coefficients of the crystal forms, and intermolecular interactions, were investigated to identify correlations with their sticking behavior. However, no obvious or consistent trends were observed, reconfirming that sticking is a complex phenomenon influenced by a variety of complex factors. These factors likely interact in ways that are not easily predictable. The results indicate that while individual properties may play a role, their combined effects, along with other potentially unaccounted environmental variables related to the method itself, contribute to the overall sticking propensity, making it difficult to isolate a single determinant.

Attachment energy morphology predictions were carried out on the systems to investigate the features of their predominant faces. Properties of the surface with the lowest attachment energy were analyzed through *Surface Analysis* using the Mercury tools. Interestingly, within the entire data set, MAN-β is the material with the highest number of hydrogen bond (HB) donors on its dominant (011) surface due to the presence of multiple hydroxyl groups on this surface termination. Similarly, LAC-I, a sugar with eight −OH groups, ranks second in the number of HB donors on its (100) surface. Most importantly, MAN-β resulted in the material with the highest number of HB donors per heavy atom, 100% of the heavy atoms present on the (011) surface being potential HB donors. Again, LAC-I falls in the second position, with 88% of its surface-heavy atoms as potential HB donors, but displays a significantly less sticky behavior. PCM-I and *p*ABA-α rank third and fourth, respectively, in terms of HB donors on their surfaces and are MSP compounds toward the high end; in contrast, the two lowest sticking materials (IBU-I and pNBA-II) do not have any HB donors on their most predominant morphological faces (see Figure 8 for more information). Potential hydrogen bonding with the chromium oxide molecules present on the stainless-steel surface or the stainless steel itself may contribute to higher sticking properties. Materials with polar groups exposed on surfaces are (in increasing order of sticking densities) pNBA-II, BA-I, MPAR-I, LAC-I, GLY-I, and MAN-β. These materials all fall into different sticking categories. Although some trends were observed, no clear linear correlation was identified between sticking and the number of HB donors on the surface, the number of HB donors per heavy atom, or the presence of polar surface groups. This suggests that these factors are only part of the many contributing factors to the complex phenomenon of sticking.

**Figure 8:**
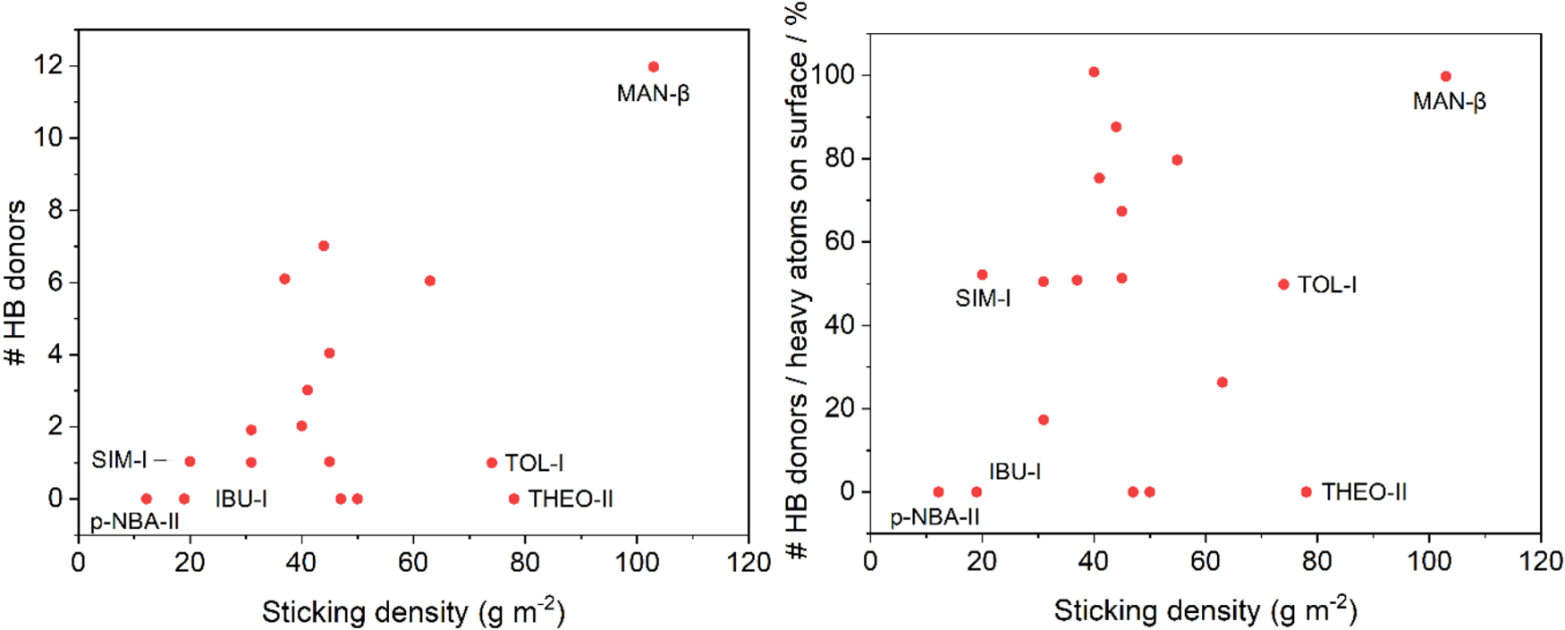
Number of hydrogen bond donors (left) and percentage of hydrogen bond donors per surface-heavy atom (right) for the set of sticky materials. The three most sticky and the three least sticky materials have been labeled.

It is noteworthy that two of the HSP materials, THEO-II and TOL-I, belong to a polar space group, with the other five polar forms (LACT_MH-I, CAF-β, LAC-I, LACT_AH-II, and MPAR-I) falling into the MSP category. Again, crystal forms belonging to a polar space group may significantly influence sticking propensities but it is not the sole determining factor.

A series of differently para-substituted benzoic acids were studied to explore how changes in functional groups affect sticking properties. *p*NBA-II (nitro group) is the least sticky material of the series, while BA-I (no functional group), *p*TA-I (methyl group), and *p*ABA-α (amino group) were categorized as MSP materials. Interestingly, *p*TA-I and *p*ABA-α exhibited the same sticking values. Although the relationship between the sticking tendencies of these materials and their molecular structure is not straightforward, it is worth noting that the poorly sticking *p*NBA-II crystals feature a closely packed interlamellar structure composed of hydrogen-bonded dimers. The packing coefficient of the crystal structure of pNBA-II at 100 K is 0.76, notably higher than those of the other benzoic acids of the series (0.71 for BA-I, 0.73 for *p*TA-I, and 0.72 for *p*ABA-α). A similar trend is observed at room temperature, with packing coefficients of 0.72 for *p*NBA-II, 0.69 for BA-I and *p*ABA-α, and 0.66 for *p*TA-I. The stronger, directional, and cohesive intermolecular interactions between molecules in the unit cells of *p*NBA-II likely account for its low tendency to form adhesive interactions with the stainless-steel surface, resulting in its low sticking density. This crystallographic feature seems to prevail over the presence of multiple polar −NO_2_ groups present on the predominant (002) surface of *p*NBA-II. While the change in functional groups clearly influences the sticking propensities of these four materials, the impact is unpredictable. Although one might anticipate a consistent trend in the sticking behavior based on the nature or position of the substituent, the data do not support such a straightforward relationship.

Our findings indicate that high sticking values partially correlate with the presence of polar surface groups, a higher number of hydrogen bond donors (including those normalized per heavy atom), and less efficient crystal packing. Although no clear or consistent trends were observed, the interplay of these factors offers valuable tools for broadly predicting the sticking categories of the materials.

## 5 Conclusions

We developed a novel, simple, and material-sparing protocol to assess the adhesion of powdered organic materials to surfaces. Using only a few hundred milligrams of material, which were stored under controlled conditions, neat grinding was performed, and the adhered material on the milling ball was quantified as a measure of the sticking tendency. After optimizing and validating the method against larger-scale techniques, it was applied to a diverse set of systems, proving to be repeatable, robust, and capable of categorizing materials by their sticking propensities.

Although no clear or consistent correlations between sticking propensities and system characteristics were found in this study, the method establishes a solid foundation for future investigations. Through collaborative efforts within the scientific community, this method will allow the building of substantial experimental data on (previously inexistent) sticking propensities of crystalline materials. By encouraging further research and data collection on diverse chemical systems, our method holds the potential to uncover trends that ultimately allow for the building of theoretical models with predictive capabilities.

Since the method uses accessible and inexpensive equipment, it is suitable for widespread adoption in many labs worldwide. The method can also be easily modified to investigate sticking propensities on other materials beyond stainless steel to suit specific research needs.

This innovative approach offers a practical tool for early detection of sticking risks in tablet manufacturing and has the potential to make a significant impact on the scientific community.

## Supplementary Material



## Notes

Figures S1–S4 providing additional characterization data and experimental observations supporting the main text, which include powder X-ray diffraction (PXRD) patterns used to monitor changes in the material form under different conditions, as well as photographic evidence from repeat experiments (PDF)
