# Supplementary material for: Simple Quantification of Sticking Propensities of Pharmaceuticals with Mechanochemistry
Source: Mol Pharm. 2025 May 12;22(6):3219–30. doi: 10.1021/acs.molpharmaceut.5c00124 (PMC12135065; doi:10.1021/acs.molpharmaceut.5c00124)
Supplement: Supplementary file 1 [file mp5c00124_si_001.pdf]

Supporting Information:

Simple Quantification of Sticking Propensities of Pharmaceuticals with Mechanochemistry

Marta Brocca[a], Helen Blade[b], Sten O. Nilsson Lill[c] and Aurora J. Cruz-Cabeza[a*]

a. Department of Chemistry, University of Durham, South Road, Durham, DH1 3LE, UK
b. Oral Product Development, Pharmaceutical Technology & Development, Operations, AstraZeneca Macclesfield, SK10 2NA, Cheshire, UK
c. Data Science and Modelling, Pharmaceutical Sciences, R&D, AstraZeneca Gothenburg, Mölndal, Sweden

*Corresponding author: aurora.j.cruz-cabeza@durham.ac.uk

Figures S1, S2 and S4 are PXRD patterns acquired on a Bruker AXS D8 Advance diffractometer (see Methods section in the main article for more info) to check for possible changes in form of the materials after storage at different relative humidities (RH) or after a neat grinding (NG) experiment. For clarity in the presentation of PXRD patterns, all intensities have been normalised.

Figure S3 shows images of milling balls in repeat experiments with materials adhered to their surfaces.

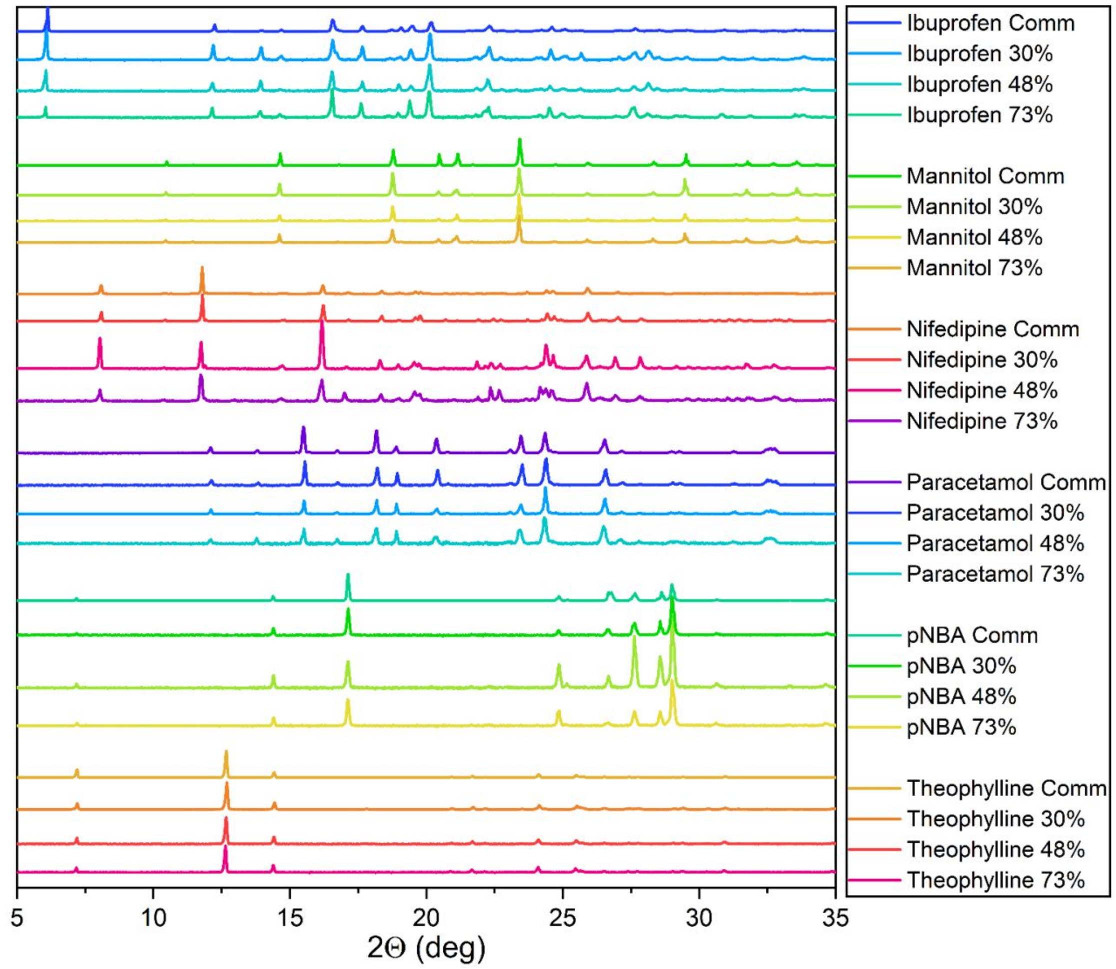

Figure S1. The effect of storage of some selected materials at different RHs. The different humidity environments were obtained with three different saturated salt solutions placed in a desiccator, in which the materials were stored for at least 2 days. PXRD spectra were taken straight after the storage; once the materials were packed on the PXRD slides, they were covered with a piece of DuPont™ Kapton® film to minimise humidity and temperature variations. Resulting spectra were compared with the commercial forms; the label "comm" stands for "commercial". No significant change in the crystal forms have been detected.

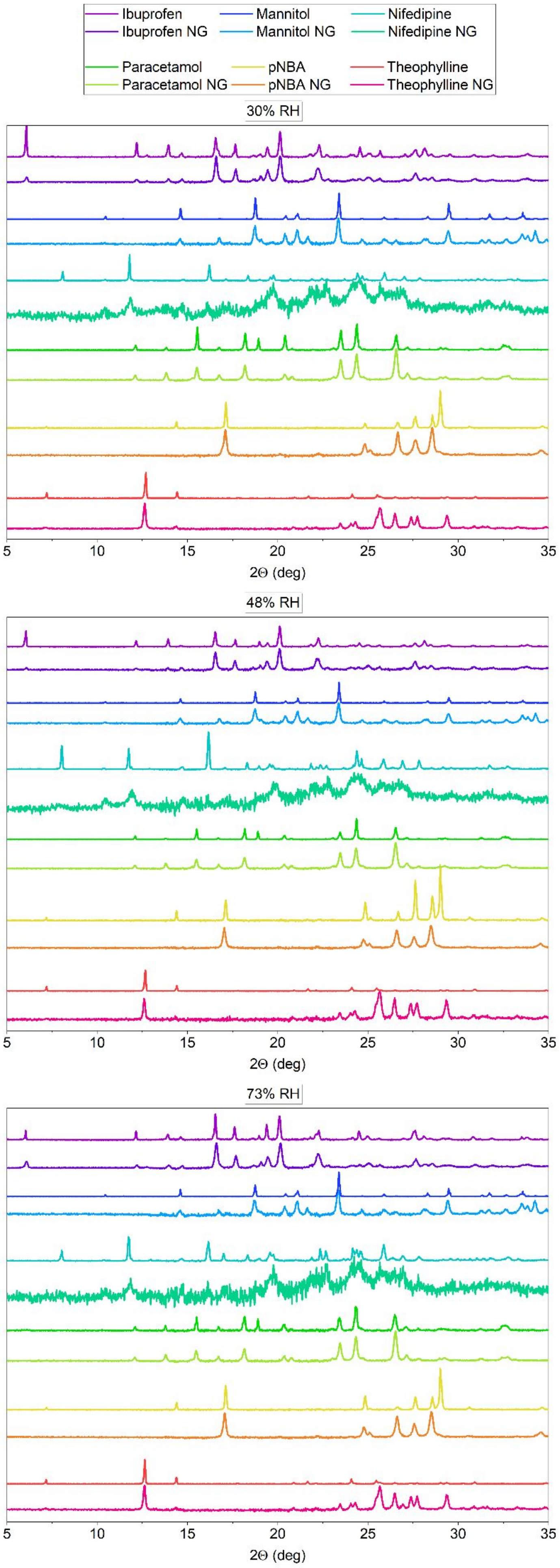

Figure S2. The effect of milling on the materials which were pre-treated at three different humidity environments. PXRD patterns of the selected systems were collected before and after the 45 min / 30 Hz NG experiment. No significant changes in form were detected apart from Nifedipine, which undergoes significant amorphisation upon milling.

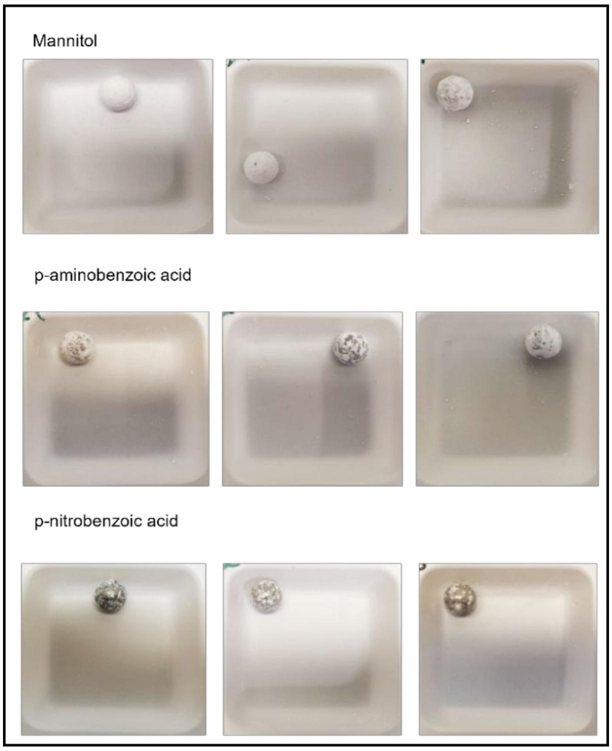

Figure S3. The repeatability of the protocol for three different systems throughout the three replicates. The figure refers to 45 min/30 Hz NG experiment before which the materials were stored at 30% RH. The figure shows that the method is repeatable throughout the measurements and that it clearly differentiates low, medium and high sticking materials.

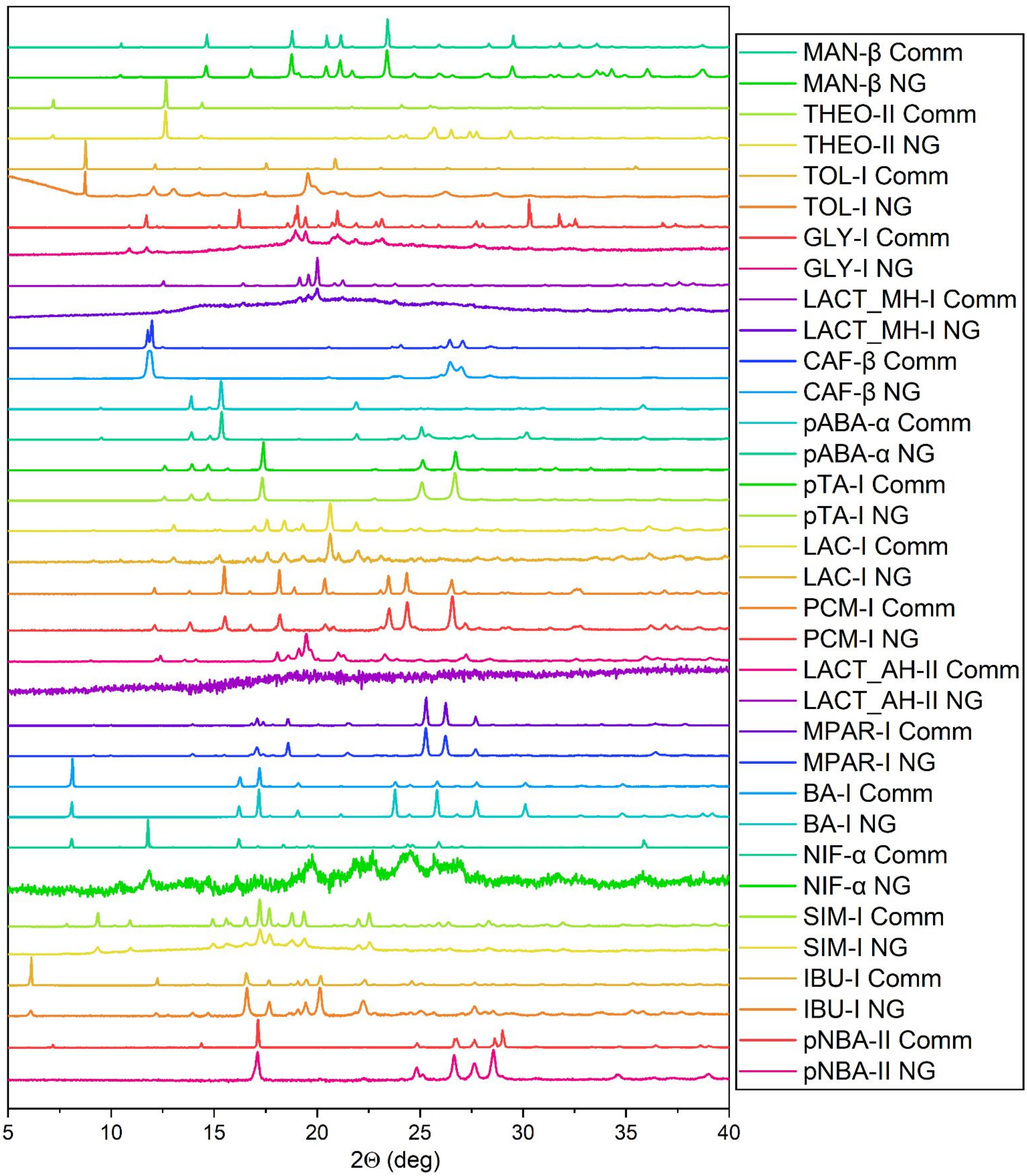

Figure S4. The effect of milling on the larger dataset of compounds. NG experiments were carried out for 45 min at 30 Hz with pre-treatment at 30% for at least 2 days. The commercial materials (labelled "comm") were analysed without any previous pre-treatment.
